# Supplementary material for: Calibration improves estimation of linkage disequilibrium on low sample sizes
Source: G3 (Bethesda). 2026 Aug 27;16(11):jkag239. doi: 10.1093/g3journal/jkag239 (PMC13732440; doi:10.1093/g3journal/jkag239)
Supplement: Supplementary file 1 [file jkag239_supplementary_data.pdf]

Supplementary material

S1 First order approximation of Pearson r^2

Let G be a genotype matrix of $n \times m$, with $G_{is} \in \{0, 1, 2\}$ for an individual $i \in \{1, \ldots, n\}$ in a genomic position $s \in \{1, \ldots, m\}$. For unphased diploid data, we only observe G_{is}, although we know that $G_{is} = H_{is}^{(1)} + H_{is}^{(2)}$, where $H_{is}^{(1)}$ and $H_{is}^{(2)}$ are the inherited parental alleles. Assume that $H_{is}^{(1)}$ and $H_{is}^{(2)}$ have a marginal distribution given by a Bernoulli with parameter p_s. In the same way, for a different position t, we define $G_{it} = H_{it}^{(1)} + H_{it}^{(2)}$, with $H_{it}^{(1)}$ and $H_{it}^{(2)}$ being Bernoullis with parameter p_t. While we assume that the pairs $H_{i\cdot}^{(1)}$ and $H_{i\cdot}^{(2)}$ are independent, we allow some dependence between $H_{is}^{(1)}$ with $H_{it}^{(1)}$ and $H_{is}^{(2)}$ with $H_{it}^{(2)}$, captured by p_{st}, the probability of having both reference alleles. Let $D_{st} = p_{st} - p_s p_t$ be the covariance between $H_{is}^{(1)}$ and $H_{it}^{(1)}$ (or equivalently the (2) haplotype).

Following the method developed by Ragsdale and Gravel (2019), where they use unbiased estimators for both the numerator and denominator of Pearson r^2, we can study the biases that we introduce by using standard estimators for D_{st}^2 and $D_{ss}D_{tt}$. This practice can be theoretically grounded by using the Taylor expansion. Assume strictly positive random variables X and Y with means μ_X and μ_Y, respectively. Let $f(X,Y) = \frac{X}{Y}$. Then, the first order Taylor expansion at (μ_X, μ_Y) is

$$\frac{X}{Y} \approx \frac{\mu_X}{\mu_Y} + (X - \mu_X)\frac{1}{\mu_Y} - (Y - \mu_Y)\frac{\mu_X}{\mu_Y^2}.$$

Hence,

$$\mathbb{E}\left[\frac{X}{Y}\right] \approx \mathbb{E}\left[\frac{\mu_X}{\mu_Y} + (X - \mu_X)\frac{1}{\mu_Y} - (Y - \mu_Y)\frac{\mu_X}{\mu_Y^2}\right] = \frac{\mu_X}{\mu_Y}.$$

Then, it makes sense to study estimators for r_{st}^2 given by

$$\widetilde{r_{st}^2} = \frac{\widetilde{D_{st}^2}}{\widetilde{D_{ss}D_{tt}}},$$

where $\widetilde{D_{st}^2}$ and $\widetilde{D_{ss}D_{tt}}$ are unbiased estimators of D_{st}^2 and $D_{ss}D_{tt}$, respectively.

Let $\widehat{D}_{st}$ be an estimator of D_{st} given by

$$\widehat{D}_{st} = \frac{1}{2(n-1)}\left(\sum_{i=1}^{n} G_{is}G_{it} - \frac{1}{n}\sum_{i,k} G_{is}G_{kt}\right).$$

The aim is to analytically compute $\mathbb{E}(\widehat{D}_{st}^2)$ and $\mathbb{E}(\widehat{D}_{ss}\widehat{D}_{tt})$ to then calculate the bias terms. To do this, let $\overline{G}_{\cdot} = \frac{1}{n}\sum_{i=1}^{n} G_{i\cdot}$.

As the computation of these expectations involves summations with up to 4 different indices, a program in the symbolic programming software `SymPy` (Meurer et al. 2017) was developed to facilitate the calculations of the following expectations.

$$\begin{aligned}\mathbb{E}[\widehat{D}_{st}] &= \frac{1}{2(n-1)}\mathbb{E}\left[\left(\sum_i G_{is}G_{it} - \frac{1}{n}\sum_{i,k} G_{is}G_{kt}\right)\right] \\ &= D_{st}.\end{aligned} \tag{S1}$$

$$\begin{aligned}\mathbb{E}[\widehat{D}_{st}\overline{G}_s] &= \frac{1}{2n(n-1)}\mathbb{E}\left[\sum_{i,j} G_{is}G_{js}G_{it} - \frac{1}{n}\sum_{i,j,k} G_{is}G_{js}G_{kt}\right] \\ &= \frac{1}{n}D_{st} + \frac{2(n-1)}{n}D_{st}p_s.\end{aligned} \tag{S2}$$

$$\begin{aligned}\mathbb{E}[\widehat{D}_{st}\overline{G}_s\overline{G}_t] &= \frac{1}{2n^2(n-1)}\mathbb{E}\left[\sum_{i,j,k} G_{is}G_{js}G_{it}G_{kt} - \frac{1}{n}\sum_{i,j,k,\ell} G_{is}G_{js}G_{kt}G_{\ell t}\right] \\ &= \frac{1}{n^2}D_{st} + \frac{2(n-1)}{n^2}D_{st}(p_s+p_t) + \frac{4(n-1)^2}{n^2}D_{st}p_sp_t + \frac{2(n-1)}{n^2}D_{st}^2.\end{aligned} \tag{S3}$$

$$\begin{aligned}\mathbb{E}[\widehat{D}_{ss}\widehat{D}_{tt}] = \frac{1}{4(n-1)^2}\mathbb{E}\Bigg[&\sum_{i,k} G_{is}^2G_{kt}^2 - \frac{1}{n}\sum_{i,k,\ell} G_{is}^2G_{kt}G_{\ell t} - \frac{1}{n}\sum_{i,j,k} G_{is}G_{js}G_{kt}^2 \\ &+ \frac{1}{n^2}\sum_{i,j,k,\ell} G_{is}G_{js}G_{kt}G_{\ell t}\Bigg] \\ = \frac{1}{2n}D_{st} - \frac{1}{n}D_{st}(p_s+p_t) &+ \frac{2}{n}D_{st}p_sp_t + \frac{n+1}{n(n-1)}D_{st}^2 + p_s(1-p_s)p_t(1-p_t).\end{aligned} \tag{S4}$$

$$\begin{aligned}\mathbb{E}[\widehat{D}_{st}^2] &= \frac{1}{4(n-1)^2}\mathbb{E}\left[\sum_{i,j} G_{is}G_{js}G_{it}G_{jt} - \frac{2}{n}\sum_{i,j,k} G_{is}G_{js}G_{it}G_{kt} + \frac{1}{n^2}\sum_{i,j,k,\ell} G_{is}G_{js}G_{kt}G_{\ell t}\right] \\ &= \frac{1}{2n}D_{st} - \frac{1}{n}D_{st}(p_s+p_t) + \frac{2}{n}D_{st}p_sp_t + \frac{n^2-n+1}{n(n-1)}D_{st}^2 + \frac{1}{n-1}p_s(1-p_s)p_t(1-p_t).\end{aligned} \tag{S5}$$

Hence, by combining (S1), (S2), (S3), (S4) and (S5), we define

$$\widetilde{D_{st}^2} = \frac{(n-1)^2}{n(n-2)}\widehat{D}_{st}^2 - \frac{n-1}{n(n-2)}\widehat{D}_{ss}\widehat{D}_{tt} - \frac{1}{2(n-1)}\widehat{D}_{st}(\overline{G}_s-1)(\overline{G}_t-1) \tag{S6}$$

and

$$\widetilde{D_{ss}D_{tt}} = -\frac{n-1}{n(n-2)}\widehat{D}_{st}^2 + \frac{(n-1)^2}{n(n-2)}\widehat{D}_{ss}\widehat{D}_{tt} - \frac{1}{2(n-1)}\widehat{D}_{st}(\overline{G}_s-1)(\overline{G}_t-1), \tag{S7}$$

which are unbiased estimators of D_{st}^2 and $D_{ss}D_{tt}$ respectively. Then, by using (S6) and (S7), the first order approximation of $\mathbb{E}\left[r_{st}^2\right]$ is given by

$$\widetilde{r_{st}^2} = \frac{\widetilde{D_{st}^2}}{\widetilde{D_{ss}D_{tt}}}. \tag{S8}$$

Then, we developed an estimator based on the Taylor expansion of r_{st}^2. In contrast to the similar estimator presented by Ragsdale and Gravel (2019), it allows the use of private data as long as we have access to the standard estimates $\widehat{D}$ and $\overline{G}$.

Furthermore, an issue with the aforementioned estimator (Ragsdale and Gravel 2019) is that there are cases where the denominator is 0. The complexity of its formulation makes the study of the roots of the denominator a difficult task. On the other hand, Lemma S1 ensures a formulation of $\widetilde{r}_{st}^2$ where we can guarantee that the estimator is well defined under some hypothesis.

Lemma S1. *Let $\widetilde{r_{st}^2}$ be defined as in* (S8)*. Then, the estimator can be written as*

$$\widetilde{r_{st}^2} = 1 + \frac{\widehat{D}_{st}^2 - \widehat{D}_{ss}\widehat{D}_{tt}}{-\frac{1}{n}\widehat{D}_{st}^2 + \frac{n-1}{n}\widehat{D}_{ss}\widehat{D}_{tt} - \frac{n-2}{2(n-1)^2}\widehat{D}_{st}(\overline{G}_s - 1)(\overline{G}_t - 1)}. \tag{S9}$$

Furthermore, for $n > 2$ and $\widehat{D}_{ss}\widehat{D}_{tt} > \frac{n^2}{4(n-1)^4}$,

$$-\frac{1}{n}\widehat{D}_{st}^2 + \frac{n-1}{n}\widehat{D}_{ss}\widehat{D}_{tt} - \frac{n-2}{2(n-1)^2}\widehat{D}_{st}(\overline{G}_s - 1)(\overline{G}_t - 1) > 0,$$

which ensures that an estimate will be well defined under this assumption.

Proof. By using that $\widetilde{D_{st}^2}$ and $\widetilde{D_{ss}D_{tt}}$ are given by (S6) and (S7), simple algebra shows that $\widetilde{r_{st}^2}$ can be written as in (S9).

For the second part of the lemma, we divide the proof by looking at the terms separately. We begin by seeing that

$$\hat{r}^2 = \frac{\widehat{D}_{st}^2}{\widehat{D}_{ss}\widehat{D}_{tt}} \iff \widehat{D}_{st}^2 = \hat{r}^2\widehat{D}_{ss}\widehat{D}_{tt}. \tag{S10}$$

Hence,

$$\begin{aligned} \frac{n-1}{n}\widehat{D}_{ss}\widehat{D}_{tt} - \frac{1}{n}\widehat{D}_{st}^2 &= \frac{n-1}{n}\widehat{D}_{ss}\widehat{D}_{tt} - \frac{1}{n}\hat{r}^2\widehat{D}_{ss}\widehat{D}_{tt} && \text{(S11)} \\ &\geq \frac{n-2}{n}\widehat{D}_{ss}\widehat{D}_{tt}. && \text{(S12)} \end{aligned}$$

By using again (S10), we have the known bound that relates the covariance with variances given by

$$|\widehat{D}_{st}| \leq \sqrt{\widehat{D}_{ss}\widehat{D}_{tt}}.$$

This implies that

$$\begin{aligned} \left|\frac{n-2}{2(n-1)^2}\widehat{D}_{st}(\overline{G}_s - 1)(\overline{G}_t - 1)\right| &\leq \frac{n-2}{2(n-1)^2}\sqrt{\widehat{D}_{ss}\widehat{D}_{tt}}\,|(\overline{G}_s - 1)(\overline{G}_t - 1)| && \text{(S13)} \\ &\leq \frac{n-2}{2(n-1)^2}\sqrt{\widehat{D}_{ss}\widehat{D}_{tt}}. && \text{(S14)} \end{aligned}$$

By using the bounds on (S11) and (S13), we get that

$$\begin{aligned} &-\frac{1}{n}\widehat{D}_{st}^2 + \frac{n-1}{n}\widehat{D}_{ss}\widehat{D}_{tt} - \frac{n-2}{2(n-1)^2}\widehat{D}_{st}(\overline{G}_s - 1)(\overline{G}_t - 1) \\ &\geq \frac{n-2}{n}\widehat{D}_{ss}\widehat{D}_{tt} - \frac{n-2}{2(n-1)^2}\sqrt{\widehat{D}_{ss}\widehat{D}_{tt}} \\ &> 0, \end{aligned}$$

where the last bound is given by using that

$$\widehat{D}_{ss}\widehat{D}_{tt} > \frac{n^2}{4(n-1)^4}.$$

□

The hypothesis of $\widehat{D}_{ss}\widehat{D}_{tt} > \frac{n^2}{4(n-1)^4}$ is easily satisfied considering that genotype data is usually preprocessed by using a minor allele frequency filter for extremely rare variants. Hence, both $\widehat{D}_{ss}$ and $\widehat{D}_{tt}$ are not allowed to be too close to zero.

S2 Calibration of sample-size aware methods

The calibration procedure presented in Equation 2 can be used for already existing methods that do a sample-size correction. To have a fairer comparison, we added a hard bound on each method, so their estimates are always between 0 and 1. Even compared to the bounded methods, the calibrated ones mostly outperform them in terms of accuracy. In particular, this improvement can be seen in the lower sample sizes. In Figure S1 we can see how calibrated methods, denoted (method) "Cal", achieve lower Root Mean Square Error (RMSE) than the hard bounded versions, denoted (method) "HB". Moreover, we can see a comparable or even improved performance against the pure "Cal", that had the best accuracy in main Figure 2. This consistent improvement by the calibration of the estimators can also be observed in Figure S2. In particular, it is worth noticing that the calibrated version of the Bulik-Sullivan approach (Bulik-Sullivan et al. 2015) appears to have the greatest performance for low n. However, for the thorough analysis in the main comparisons we use the basic sample calibration. This is to avoid p-hacking by selecting the top performer, as we are comparing many alternatives, but it is worth mentioning that there might be an even better method by calibrating sample-size-aware estimates.

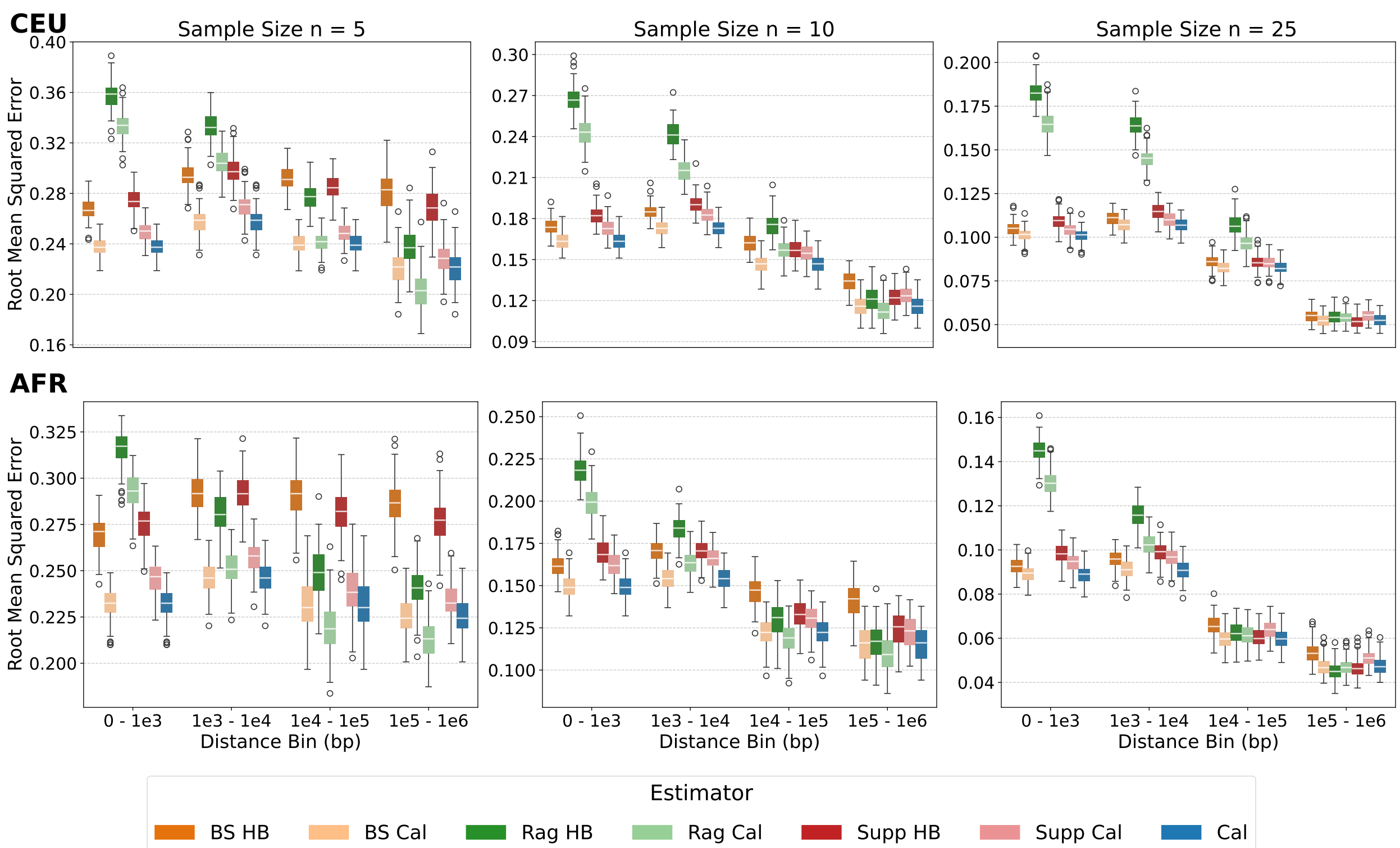

Figure S1: Distribution of Root Mean Squared Error (RMSE) for r^2 estimators across genomic distance and sample size. Each row is computed by using different data (CEU and AFR), while each of the three columns corresponds to a different subsample size (n). The x-axis in each plot represents genomic distance, divided into bins. Within each bin, the boxplots show the distribution of RMSE values obtained from 100 independent bootstrap replicates, where each color represents a different r^2 estimator.

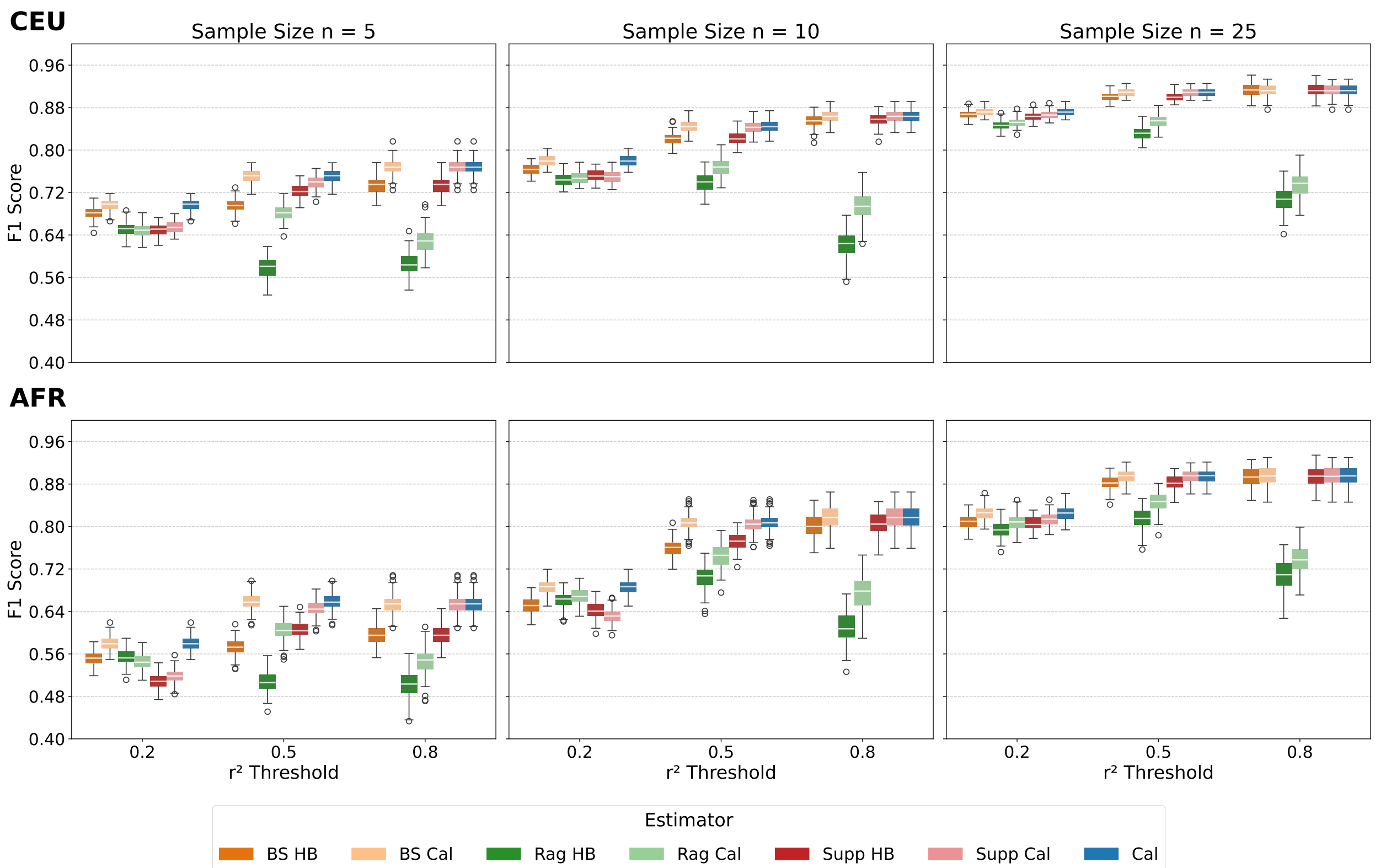

Figure S2: F1 Score for LD pruning performance, compared across populations and sample sizes. The top and bottom rows show performance on data from the CEU and AFR populations, respectively. Each column displays results for a different subsample size (n). The x-axis represents the r^2 threshold for pruning, while the y-axis shows the F1 score, where a higher value indicates a better balance between precision (avoiding over-pruning) and recall (avoiding under-pruning). The boxplots are based on the resulting F1 score for each method over 100 independent bootstrap replicates.

S3 Computation of LD Scores

To further investigate the implications of having more accurate estimates of r^2, we measured the Root Mean Square Error (RMSE) of each method when computing LD scores. The LD score of a variant summarizes the amount of genetic variation it tags by aggregating its correlations (r^2) with neighboring variants (Bulik-Sullivan et al. 2015). For a variant j, the LD score is defined as

$$\ell_j := \sum_{i \in I(j)} r_{ij}^2,$$

where $I(j)$ is the set of variants within a 500,000 bp window centered on variant j. To estimate the RMSE, we subsampled our datasets with $n = 5, 10, 25$ individuals. From each subsample, we randomly selected 1000 variants, estimated their LD scores, and compared them to the "true" scores obtained using the full dataset. This procedure was repeated 50 times on independent subsamples and target variants, and the RMSE was computed for each method.

Figure S3 presents the boxplots of the RMSE over the 50 replicates. As expected, the one-step calibration method "Cal" does not yield accurate LD score estimates. This is due to the fact that, by only admitting values between 0 and 1, the upward bias, although heavily reduced, is still there. This issue is particularly relevant in metrics where the total LD score is more important than the accuracy of individual pairwise estimates, i.e., when bias dominates over variance. In contrast, the sample-aware estimators ("BS", "Rag", and "Supp") perform substantially better, with "BS" often showing a slight advantage over the others. The best performance, however, is consistently achieved by the two-step calibration method ("mCal"), which outperforms all other approaches, especially at smaller sample sizes ($n = 5$ and $n = 10$). This improvement arises because "mCal" trades a small loss in accuracy on dependent variant pairs for a much stronger reduction in bias on independent pairs, leading to overall more reliable LD score estimates.

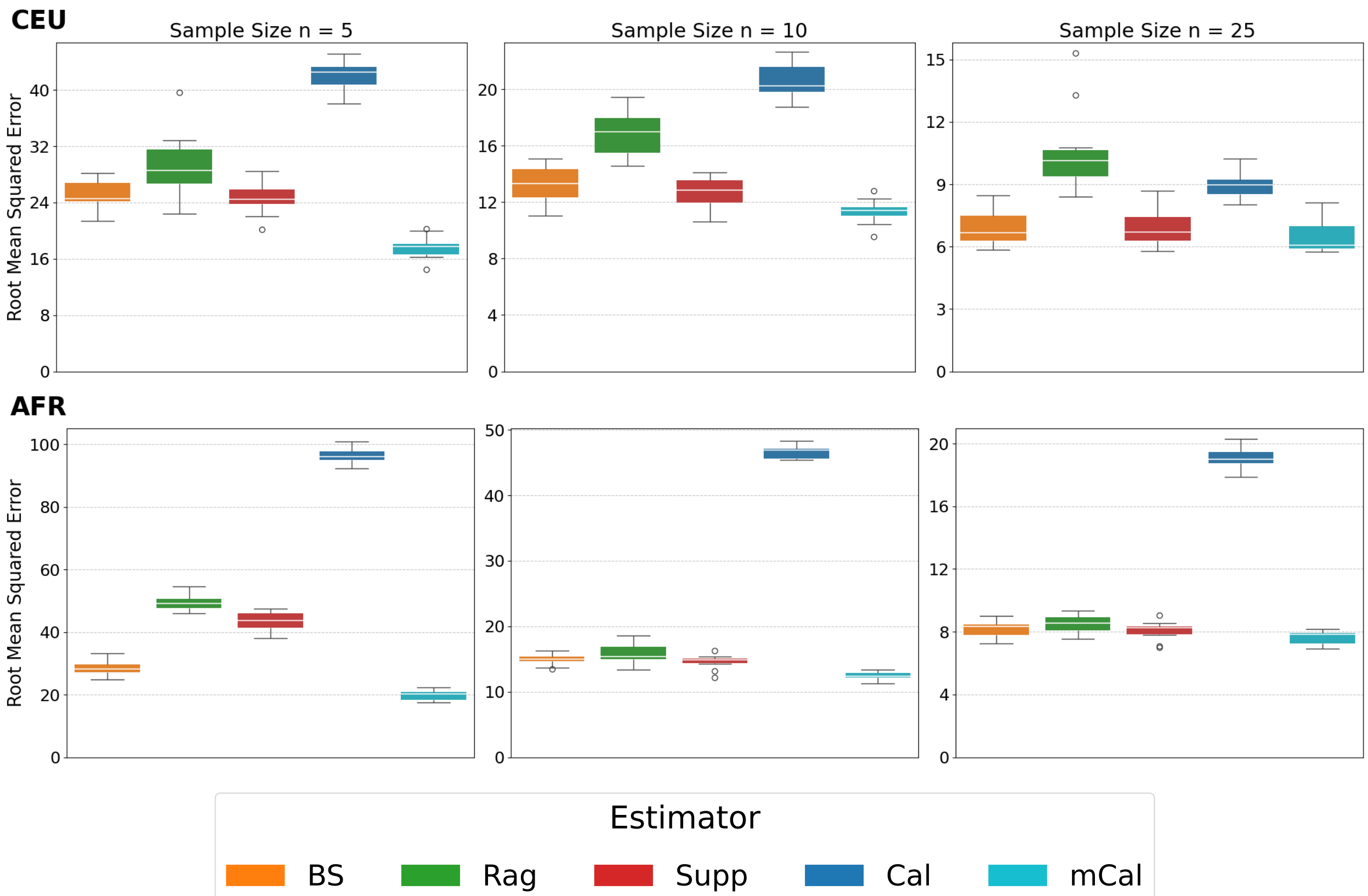

Figure S3: Root Mean Squared Error (RMSE) for LD score estimation, compared across populations and sample sizes. The top and bottom rows show results for the CEU and AFR populations, respectively. Each panel corresponds to a different subsample size (n). The y-axis shows the RMSE of the estimated LD scores relative to the true values obtained from the full sample. Each RMSE value is computed over the LD scores of 1,000 randomly chosen SNPs. The boxplots summarize results across 50 independent replicates, each based on random subsamples of individuals and variants.

S4 Calibration for pseudohaploid data

The sample sizes that motivate this work are smallest in ancient DNA, where the genotypes can come from pseudohaploid data: instead of having a diploid genotype, a single read is drawn at each site, so the observed data are $G_{is} \in \{0, 1\}$. Sampling one allele per site halves the covariance between the two loci while leaving the variances of the single alleles unchanged, so the squared correlation is attenuated by a factor of four. The consequence is that the diploid curves do not merely lose accuracy, they correct in the wrong direction, since the diploid r^2 is biased upwards with respect to ρ^2 while the pseudohaploid one is biased downwards.

Nevertheless, the calibration itself does not depend on the data being diploid, only on being able to simulate the sampling process. It is therefore enough to generate the genotype matrices under the pseudohaploid model and rebuild the curves. We compare four estimators: the naive $4r^2$, which rescales the pseudohaploid statistic to correct the fourfold attenuation; the same estimator hard-bounded to $[0, 1]$, denoted $4r^2$ (HB); and the one- and two-step calibrations built under the pseudohaploid model.

We use the bootstrap procedure of the main text: for each of 100 replicates we draw a subsample of n individuals, sample one allele per site, and estimate r^2 for pairs stratified into genomic distance bins. The ground truth is the standard r^2 computed on the full, diploid dataset, so the estimators are asked to recover the diploid population value from pseudohaploid data.

The naive $4r^2$ fails: at $n = 5$ its bias is close to 1 in every distance bin, since the pseudohaploid r^2 is itself strongly biased upwards at this sample size and the rescaling multiplies that bias by four. It is omitted from Figures S4 and S5 for scale. Bounding it to $[0, 1]$ helps, but a substantial bias remains, reaching 0.60 in the 100kb to 1Mb bin at $n = 5$ in both datasets. The calibrations do considerably better: in the same bin the one-step calibration has a bias of 0.20 and the two-step calibration of -0.014, and the two-step calibration is close to unbiased for distant pairs at every sample size. The same ordering holds for the RMSE, where the one-step calibration has a lower RMSE than $4r^2$ (HB) in 22 of the 24 combinations of dataset, sample size and distance bin; at $n = 25$ in the 100kb to 1Mb bin its RMSE is 0.13, against 0.26 for $4r^2$ (HB).

The errors nevertheless remain large at the smallest sample sizes: at $n = 5$ the RMSE of the calibrated estimators is around 0.4, roughly twice what it is for diploid data. This is a limit of the data rather than of the calibration, since sampling a single allele per site discards half of the observations. The calibration is therefore the best of the available options for this data type, but it does not make extremely small pseudohaploid samples, such as $n = 5$, informative.

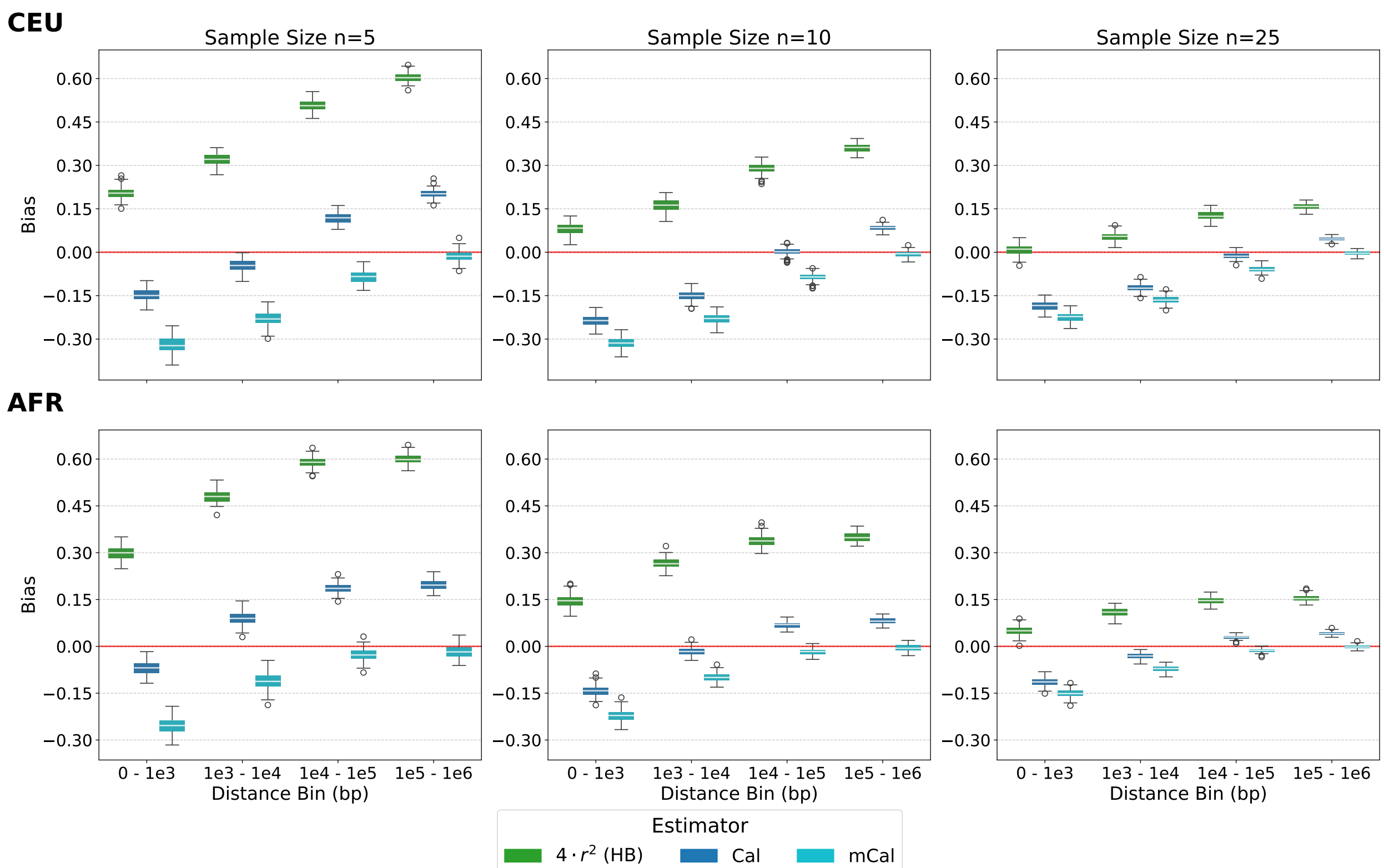

Figure S4: Bias of the estimators of r^2 under the pseudohaploid model, as a function of distance and sample size. Each row is computed by using different data (CEU and AFR), while each of the three columns corresponds to a different subsample size (n). The x-axis represents genomic distance, divided into bins. Within each bin, the boxplots show the distribution of the bias obtained from 100 independent bootstrap replicates, computed against the standard r^2 of the full diploid dataset, where each color represents a different estimator. The naive $4r^2$ is omitted for scale, as its bias is close to 1 at $n = 5$.

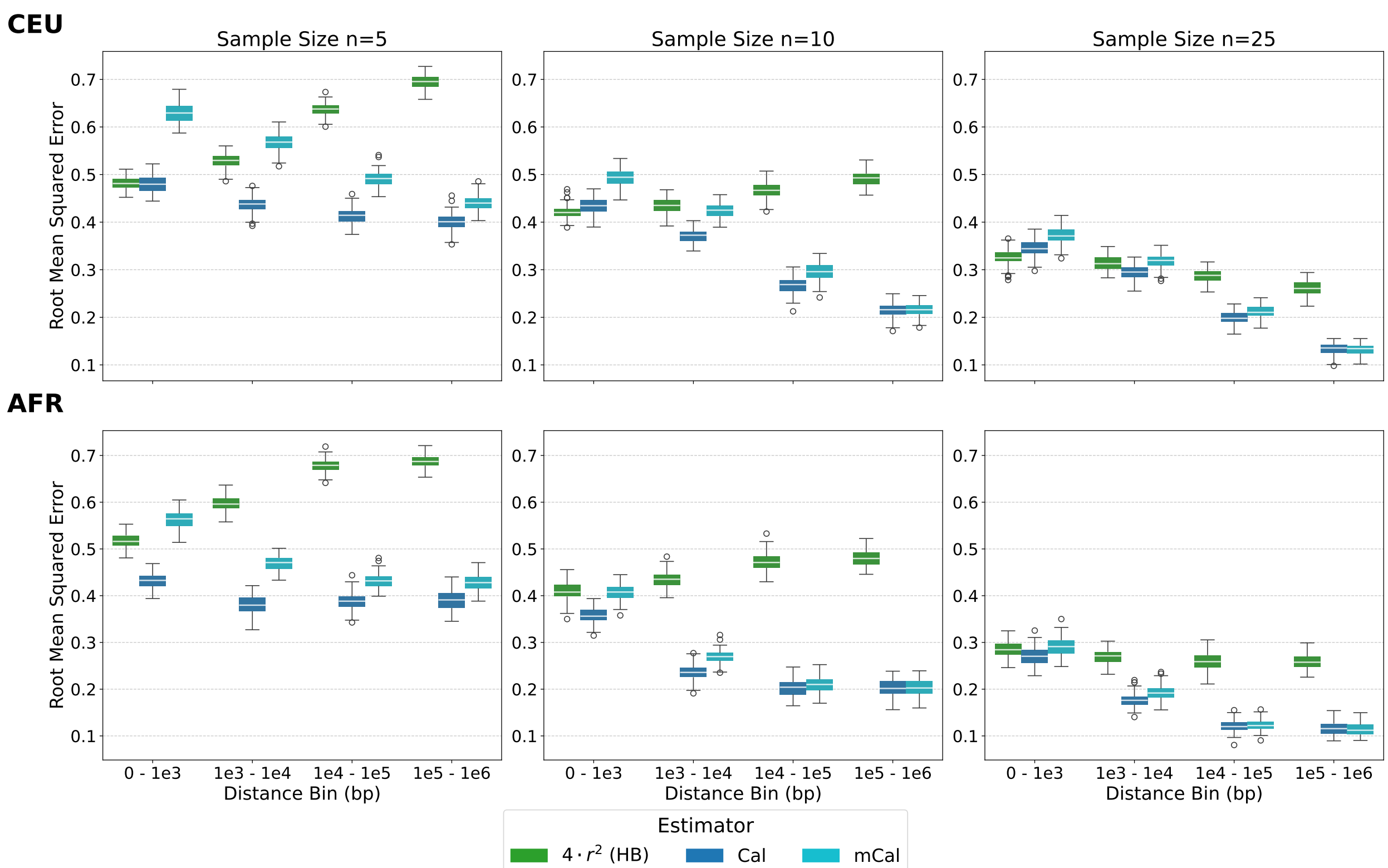

Figure S5: Distribution of RMSE for the estimators of r^2 under the pseudohaploid model, with the same layout as Figure S4.

S5 Calibration of an LD matrix without the genotypes

The calibration described so far takes a genotype matrix as input. In practice, LD is often distributed as a precomputed matrix rather than as genotypes, either because the individual data are not shareable or because the matrix is used as a reference panel. Here we show that the calibration only requires the LD matrix and the sample size, and that it can therefore be applied to a matrix that has already been corrected for something else. We take as an example the LD measure adjusted for population structure of Bercovich et al. (2025), which addresses a bias that our calibration does not.

The two biases are of a different nature. The bias caused by the sample size vanishes as n grows, while the one caused by population structure does not: when individuals from different populations are pooled, the LD of the pooled population is genuinely larger, as described by the two-locus Wahlund effect. The adjusted measure of Bercovich et al. (2025) removes the second by correlating the genotype residuals obtained after projecting out the top k principal components,

$$R = G - 2\widehat{\Pi}, \qquad \widehat{D}_{adj} = \frac{R^\top (I - J/n) R}{2(n-k)}, \qquad (r^2_{adj})_{st} = \frac{(\widehat{D}_{adj})^2_{st}}{(\widehat{D}_{adj})_{ss}(\widehat{D}_{adj})_{tt}},$$

where $\widehat{\Pi}$ is the matrix of individual-specific allele frequency estimates obtained from the top k principal components, I is the $n \times n$ identity matrix, and J is the $n \times n$ matrix of ones. This leaves the sample size bias untouched, since r^2_{adj} is still a ratio of estimated covariances.

Calibrating r^2_{adj} requires the sample allele frequencies and the sample size, and neither is obviously available without the genotype data. Both, however, can be read off the LD matrix itself. For the sample size, the residuals lie in a space of dimension $n-k$, since k degrees of freedom are spent on the projection, so the calibration uses an effective sample size $n_{\text{eff}} = n - k$. For the frequencies, the pooled heterozygosity of a site is the sum of a within-population and a between-population component, and the projection removes the second, so only the first survives in the residual. We therefore define an effective frequency $\widetilde{p}_s$ as the frequency of the homogeneous population whose heterozygosity matches the residual variance,

$$\widetilde{p}_s(1 - \widetilde{p}_s) = (\widehat{D}_{adj})_{ss},$$

which is exactly the diagonal of the LD matrix. The calibration is then the one already described, applied to r^2_{adj} at the pair $(\widetilde{p}_s, \widetilde{p}_t)$ and at n_{eff}, using the same precomputed curves as in the main text. No new simulation is needed, and the genotypes are never used: the input is $\widehat{D}_{adj}$ and n.

Since $\widetilde{p}$ is obtained by inverting $p(1-p)$, it assumes Hardy–Weinberg equilibrium and it is unreliable where the residual variance is small. We therefore discard pairs with $\widetilde{p} < 0.05$, a filter that can also be applied without the genotypes and that removes both the effectively rare variants and the sites strongly departing from equilibrium.

We evaluate this on the structured human dataset of Bercovich et al. (2025): 50 CEU, 50 YRI and 50 ASW individuals from the 1000 Genomes Project (1000 Genomes Project Consortium 2015), chromosome 22, restricted to variants with a minor allele frequency above 5% (129,688 variants). The ASW individuals are admixed between European and African ancestry, so the sample contains two ancestral populations and one admixed group. A single principal component ($k = 1$) is enough to capture this structure. The adjusted measure therefore removes the structure and leaves precisely the bias that the calibration targets.

We then draw 100 random subsamples for each of two sizes: 16 individuals (5 CEU, 5 YRI and 6 ASW) and 26 individuals (9 CEU, 9 YRI and 8 ASW), which with $k = 1$ give $n_{\text{eff}} = 15$ and

$n_{\text{eff}} = 25$ respectively. For each subsample the principal component is refitted, so that everything the estimators use comes from the subsample itself. The ground truth is the adjusted r^2 computed on all 150 individuals. As before, pairs are stratified into genomic distance bins, with up to 400 pairs per bin.

Figure S6 shows the bias and the RMSE against this reference for the adjusted r^2 without any sample-size correction ("Samp"), and after the one-step ("Cal") and two-step ("mCal") calibrations. The uncorrected adjusted r^2 is biased upwards, and increasingly so with distance, since distant pairs are close to independence: at $n = 16$ its bias grows from 0.036 in the 0–1 kb bin to 0.066 in the 100 kb–1 Mb bin. The calibration removes most of it. In the 100 kb–1 Mb bin at $n = 16$, the bias falls to 0.023 with the one-step and to -0.007 with the two-step calibration, while the RMSE drops from 0.117 to 0.076 and 0.075. The same pattern holds at $n = 26$, with smaller differences, as there is less bias to remove. As in the main text, the two calibrations are accurate in complementary regions, the one-step being preferable for closely linked pairs and the two-step for the distant ones. All of this is obtained from the adjusted matrix and the sample size alone.

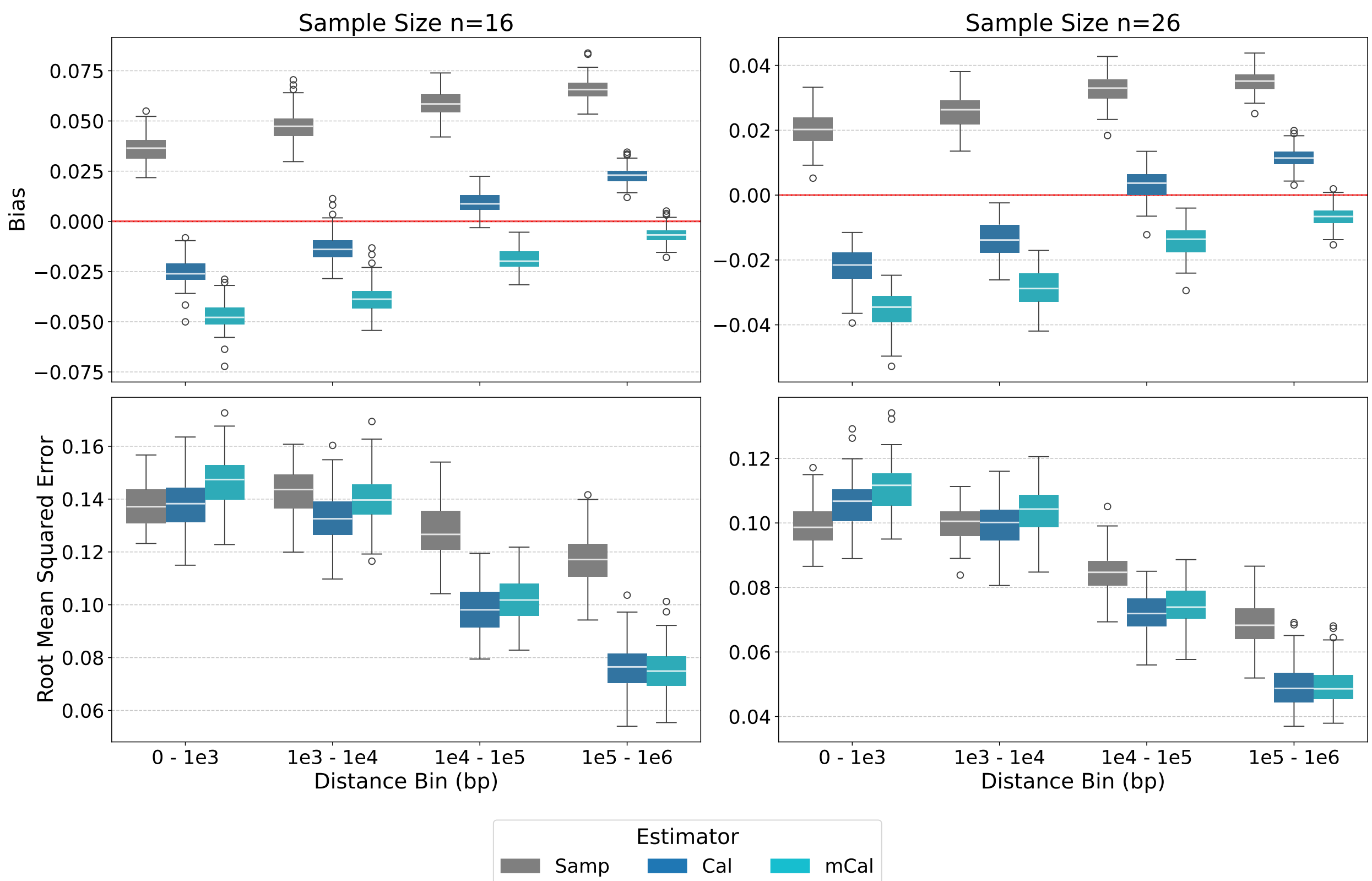

Figure S6: Bias (top) and Root Mean Squared Error (bottom) of the adjusted r^2 of Bercovich et al. (2025) without a sample-size correction ("Samp") and after the one-step ("Cal") and two-step ("mCal") calibrations, on subsamples from the 1000 Genomes Project. Each column corresponds to a different subsample size: 16 individuals (5 CEU, 5 YRI, 6 ASW) and 26 individuals (9 CEU, 9 YRI, 8 ASW). The x-axis represents genomic distance, divided into bins. Within each bin, the boxplots show the distribution of the corresponding metric over 100 independent subsamples, computed against the adjusted r^2 of the full sample of 150 individuals, where each color represents a different estimator. The calibration uses only the adjusted LD matrix and the sample size.

Supplementary Algorithms

Algorithm S1 Estimation of $g_{p_s,p_t}(\rho^2)$

Input: Sample size n, discretized grid of ρ^2 values between 0 and 1 ($\texttt{grid\_}\rho^2$), number of repetitions $\texttt{Nrep}$.

Output: Estimated map $\widehat{g}_{(p_s,p_t)}(\rho^2)$.

for allele frequencies $(p_s, p_t) \in \{1/(2n), \dots, (2n-1)/(2n)\}^2$ **do**
  for each ρ^2 in $\texttt{grid\_}\rho^2$ **do**
    if (p_s, p_t, ρ^2) is a valid tuple **then** ▷ satisfies D bounds
      for rep $= 1, \dots, \texttt{Nrep}$ **do**
        repeat
          Simulate genotype matrix $G \sim (n, p_s, p_t, \rho^2)$
        until $\text{Var}(G_{\cdot s}) > 0$ and $\text{Var}(G_{\cdot t}) > 0$ ▷ r^2 has to be well defined
        Compute $r^2_{\texttt{rep}} \leftarrow r^2(G)$ ▷ following Equation (1)
      end for
      $\widehat{g}_{(p_s,p_t)}(\rho^2) \leftarrow \frac{1}{\texttt{Nrep}} \sum_{\texttt{rep}=1}^{\texttt{Nrep}} r^2_{\texttt{rep}}$
    end if
  end for
end for

Algorithm S2 Calibration of observed r^2 using $\widehat{g}$

Input: Two-locus genotype matrix G, precomputed map $\widehat{g}$.

Output: Calibrated estimate $\widehat{r^2_{st}}$.

$(\widehat{p}_s, \widehat{p}_t) \leftarrow \frac{1}{2n} \left(\sum_{i=1}^{n} G_{is}, \sum_{i=1}^{n} G_{it}\right)$ ▷ estimate allele frequencies
Compute $r^2_{st} \leftarrow r^2(G_{\cdot s}, G_{\cdot t})$ ▷ following Equation (1)
$\widehat{r^2_{st}} \leftarrow \widehat{g}^{-1}_{(\widehat{p}_s,\widehat{p}_t)}(r^2_{st})$ ▷ calibrate via inverse lookup/interpolation

Supplementary Figures

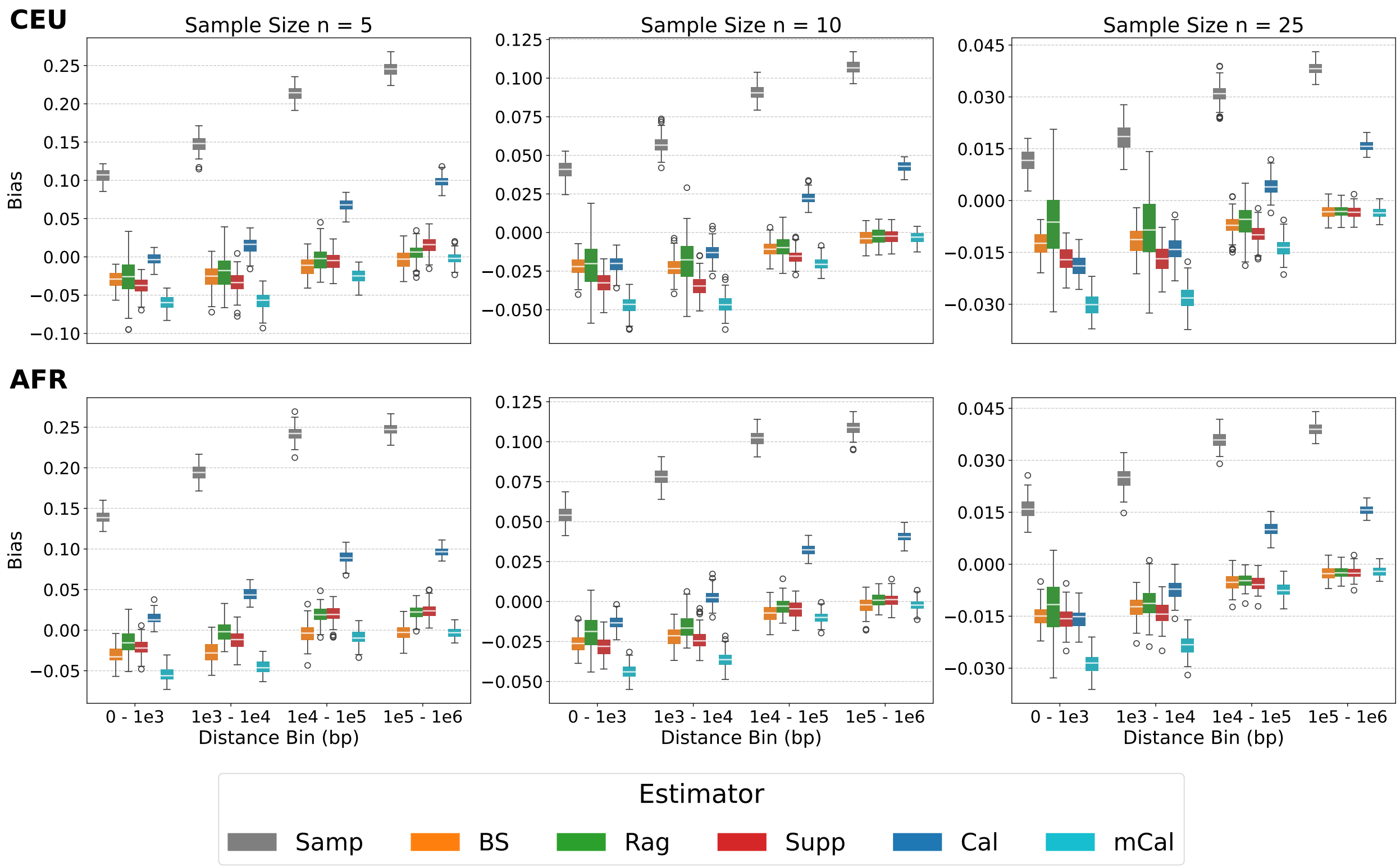

Figure S7: Bias of the estimates as a function of distance and sample size. Each row displays the distributions for different sample sizes. The x-axis shows genomic distance bins. The boxplots within each bin represent the distribution of the bias calculated over 100 bootstrap replicates.

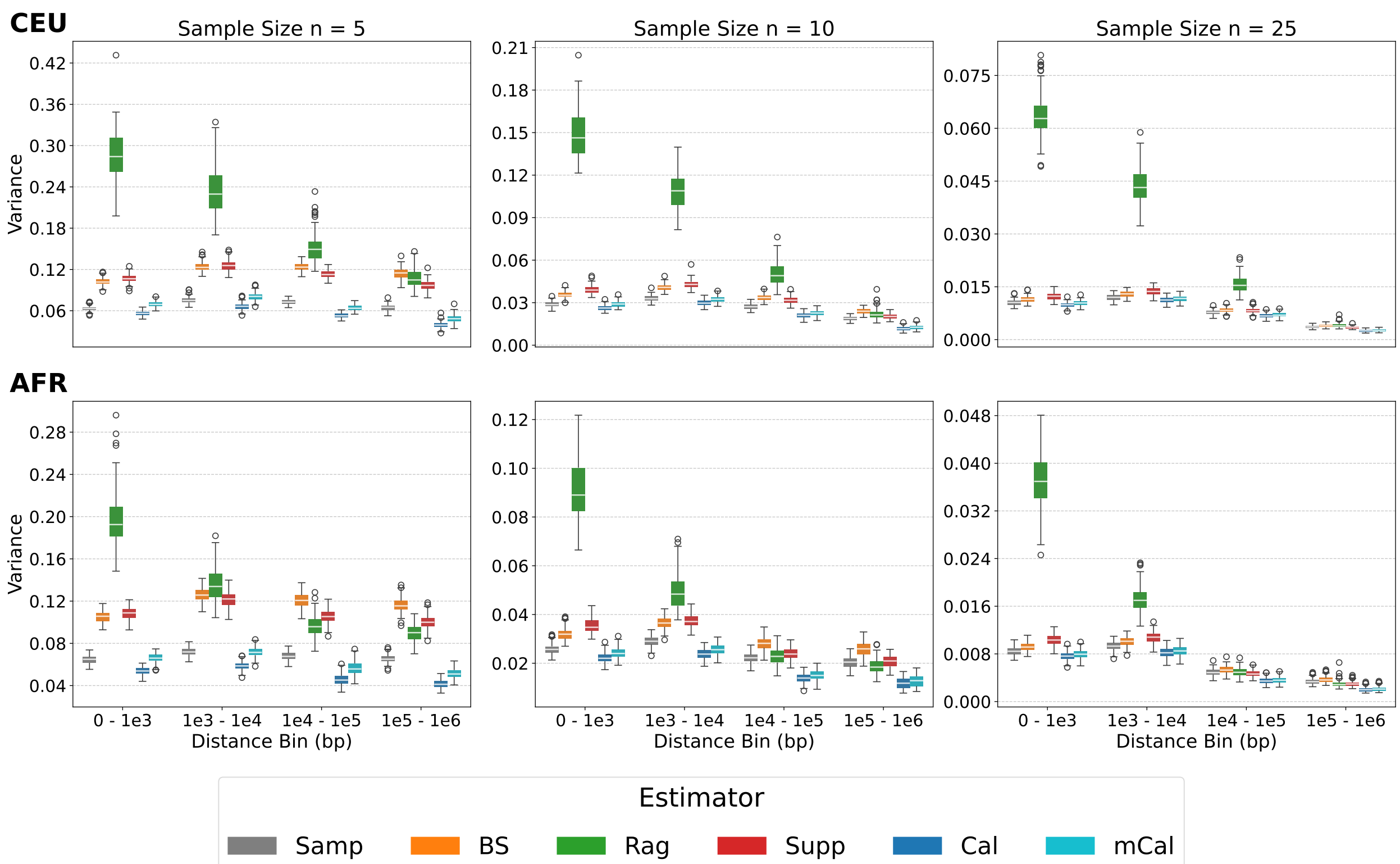

Figure S8: Variances of the errors of the estimates as a function of distance and sample size. Each row displays the distributions for different sample sizes. The x-axis shows genomic distance bins. The boxplots within each bin represent the distribution of the variance of the errors calculated over 100 bootstrap replicates.

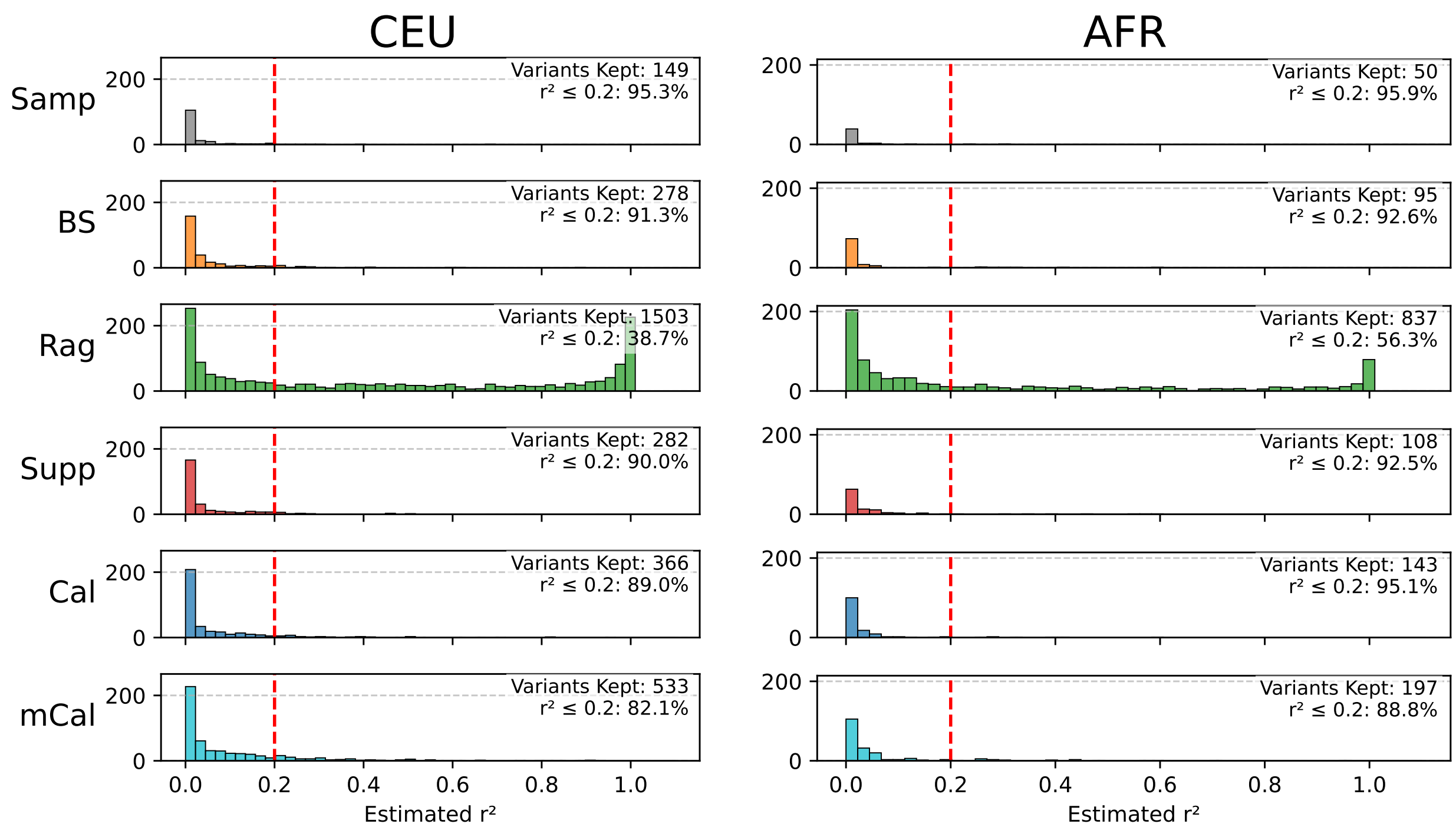

Figure S9: Distribution of r^2 for adjacent SNPs remaining after LD pruning. The analysis was performed on a sample of $n = 5$ individuals. The left panel shows results from the 1000 Genomes CEU population, while the right panel shows results from the simulated AFR population. Each row displays the histogram of the true r^2 values for the set of SNPs kept by each estimator. The red dashed line indicates the pruning threshold ($r^2 = 0.2$). The annotations in each subplot provide the total number of variants kept and the percentage of remaining adjacent pairs with $r^2 \leq 0.2$.

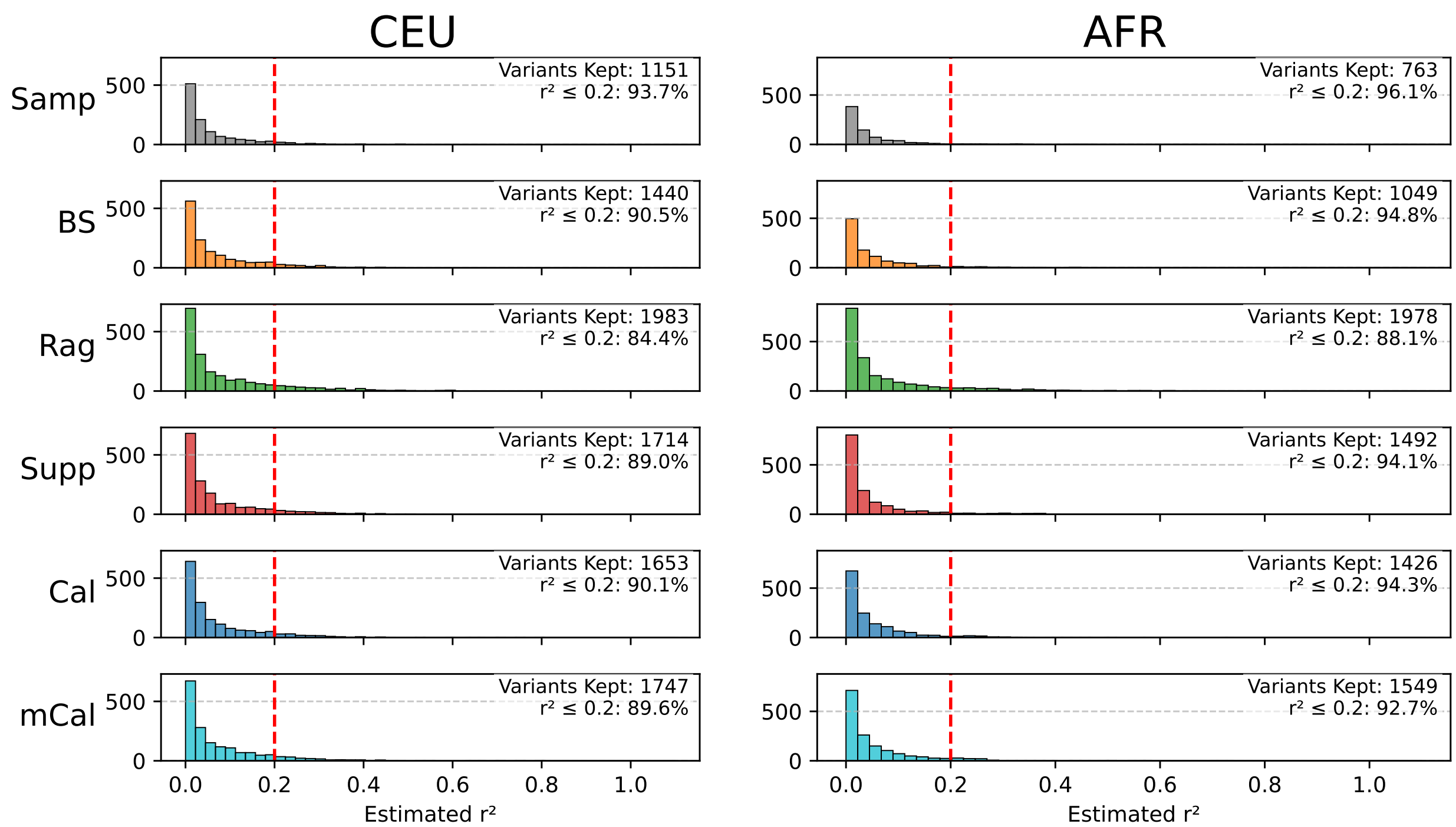

Figure S10: Distribution of r^2 for adjacent SNPs remaining after LD pruning. The analysis was performed on a sample of $n = 25$ individuals. The left panel shows results from the 1000 Genomes CEU population, while the right panel shows results from the simulated AFR population. Each row displays the histogram of the true r^2 values for the set of SNPs kept by each estimator. The red dashed line indicates the pruning threshold ($r^2 = 0.2$). The annotations in each subplot provide the total number of variants kept and the percentage of remaining adjacent pairs with $r^2 \leq 0.2$.

Supplementary Tables

**Bin Range (bp)**	**Available Pairs**
[0, 1,000)	9,769
[1,000, 10,000)	73,895
[10,000, 100,000)	656,239
[100,000, 1,000,000)	5,858,331

Table S1: Number of available SNP pairs per distance bin. Analysis was performed on the CEU dataset, which corresponds to a genomic region of Chromosome 22 containing 14,465 SNPs from the 1000 Genomes Project.

**Bin Range (bp)**	**Available Pairs**
[0, 1,000)	20,229
[1,000, 10,000)	171,386
[10,000, 100,000)	1,645,073
[[100,000, 1,000,000)	15,637,545

Table S2: Number of available SNP pairs per distance bin. Analysis was performed on the AFR dataset, which corresponds to a genomic region of Chromosome 22 containing 13,543 SNPs from a simulated African population.

	Bias		Variance		RMSE	
**Estimator**	**Mean**	**Std Dev**	**Mean**	**Std Dev**	**Mean**	**Std Dev**
**Sample Size n = 5**						
Samp	1.78×10^{-1}	5.55×10^{-2}	6.90×10^{-2}	6.56×10^{-3}	3.21×10^{-1}	3.30×10^{-2}
BS	-1.77×10^{-2}	1.64×10^{-2}	1.16×10^{-1}	1.11×10^{-2}	3.41×10^{-1}	1.61×10^{-2}
Rag	-1.06×10^{-2}	2.32×10^{-2}	1.94×10^{-1}	7.46×10^{-2}	4.33×10^{-1}	8.56×10^{-2}
Supp	-1.53×10^{-2}	2.50×10^{-2}	1.11×10^{-1}	1.24×10^{-2}	3.33×10^{-1}	1.89×10^{-2}
Cal	4.47×10^{-2}	4.12×10^{-2}	5.38×10^{-2}	1.05×10^{-2}	2.39×10^{-1}	1.65×10^{-2}
mCal	-3.50×10^{-2}	2.56×10^{-2}	6.57×10^{-2}	1.26×10^{-2}	2.59×10^{-1}	2.72×10^{-2}
**Sample Size n = 10**						
Samp	7.38×10^{-2}	2.69×10^{-2}	2.69×10^{-2}	5.45×10^{-3}	1.82×10^{-1}	9.89×10^{-3}
BS	-1.49×10^{-2}	1.01×10^{-2}	3.34×10^{-2}	6.49×10^{-3}	1.83×10^{-1}	1.87×10^{-2}
Rag	-1.24×10^{-2}	1.39×10^{-2}	8.25×10^{-2}	5.08×10^{-2}	2.72×10^{-1}	9.42×10^{-2}
Supp	-2.15×10^{-2}	1.46×10^{-2}	3.36×10^{-2}	9.09×10^{-3}	1.83×10^{-1}	2.71×10^{-2}
Cal	7.78×10^{-3}	2.63×10^{-2}	2.23×10^{-2}	7.11×10^{-3}	1.50×10^{-1}	2.27×10^{-2}
mCal	-2.96×10^{-2}	1.92×10^{-2}	2.43×10^{-2}	7.81×10^{-3}	1.57×10^{-1}	2.92×10^{-2}
**Sample Size n = 25**						
Samp	2.48×10^{-2}	1.09×10^{-2}	8.44×10^{-3}	3.28×10^{-3}	9.45×10^{-2}	1.55×10^{-2}
BS	-8.54×10^{-3}	4.97×10^{-3}	9.13×10^{-3}	3.54×10^{-3}	9.39×10^{-2}	2.04×10^{-2}
Rag	-5.89×10^{-3}	7.77×10^{-3}	3.18×10^{-2}	2.39×10^{-2}	1.63×10^{-1}	7.40×10^{-2}
Supp	-1.18×10^{-2}	6.58×10^{-3}	9.44×10^{-3}	4.06×10^{-3}	9.52×10^{-2}	2.34×10^{-2}
Cal	-3.28×10^{-3}	1.43×10^{-2}	7.60×10^{-3}	3.41×10^{-3}	8.57×10^{-2}	2.15×10^{-2}
mCal	-1.88×10^{-2}	1.14×10^{-2}	7.90×10^{-3}	3.56×10^{-3}	8.84×10^{-2}	2.39×10^{-2}

Table S3: Performance metrics (mean, standard deviation and RMSE) for r^2 estimators across different sample sizes in the CEU dataset.

	Bias		Variance		RMSE	
**Estimator**	**Mean**	**Std Dev**	**Mean**	**Std Dev**	**Mean**	**Std Dev**
**Sample Size n = 5**						
Samp	2.06×10^{-1}	4.43×10^{-2}	6.75×10^{-2}	4.92×10^{-3}	3.33×10^{-1}	2.87×10^{-2}
BS	-1.65×10^{-2}	1.72×10^{-2}	1.17×10^{-1}	1.02×10^{-2}	3.43×10^{-1}	1.45×10^{-2}
Rag	6.58×10^{-3}	1.92×10^{-2}	1.30×10^{-1}	4.57×10^{-2}	3.56×10^{-1}	6.04×10^{-2}
Supp	2.54×10^{-3}	2.22×10^{-2}	1.09×10^{-1}	1.02×10^{-2}	3.30×10^{-1}	1.53×10^{-2}
Cal	6.12×10^{-2}	3.43×10^{-2}	5.00×10^{-2}	7.88×10^{-3}	2.34×10^{-1}	1.33×10^{-2}
mCal	-2.68×10^{-2}	2.39×10^{-2}	6.13×10^{-2}	9.42×10^{-3}	2.49×10^{-1}	2.06×10^{-2}
**Sample Size n = 10**						
Samp	8.59×10^{-2}	2.21×10^{-2}	2.43×10^{-2}	3.99×10^{-3}	1.79×10^{-1}	1.00×10^{-2}
BS	-1.43×10^{-2}	1.13×10^{-2}	3.06×10^{-2}	4.89×10^{-3}	1.75×10^{-1}	1.44×10^{-2}
Rag	-8.89×10^{-3}	1.14×10^{-2}	4.56×10^{-2}	3.00×10^{-2}	2.03×10^{-1}	6.78×10^{-2}
Supp	-1.40×10^{-2}	1.34×10^{-2}	2.93×10^{-2}	7.67×10^{-3}	1.71×10^{-1}	2.35×10^{-2}
Cal	1.56×10^{-2}	2.23×10^{-2}	1.79×10^{-2}	5.60×10^{-3}	1.35×10^{-1}	1.89×10^{-2}
mCal	-2.36×10^{-2}	1.80×10^{-2}	1.94×10^{-2}	6.05×10^{-3}	1.40×10^{-1}	2.44×10^{-2}
**Sample Size n = 25**						
Samp	2.90×10^{-2}	9.62×10^{-3}	6.50×10^{-3}	2.52×10^{-3}	8.53×10^{-2}	1.24×10^{-2}
BS	-8.73×10^{-3}	5.85×10^{-3}	7.06×10^{-3}	2.73×10^{-3}	8.29×10^{-2}	1.72×10^{-2}
Rag	-7.53×10^{-3}	6.16×10^{-3}	1.55×10^{-2}	1.38×10^{-2}	1.12×10^{-1}	5.52×10^{-2}
Supp	-9.53×10^{-3}	6.35×10^{-3}	7.20×10^{-3}	3.50×10^{-3}	8.27×10^{-2}	2.21×10^{-2}
Cal	7.49×10^{-4}	1.29×10^{-2}	5.34×10^{-3}	2.71×10^{-3}	7.17×10^{-2}	1.91×10^{-2}
mCal	-1.54×10^{-2}	1.13×10^{-2}	5.54×10^{-3}	2.80×10^{-3}	7.38×10^{-2}	2.16×10^{-2}

Table S4: Performance metrics (mean, standard deviation and RMSE) for r^2 estimators across different sample sizes in the AFR dataset.

References

1000 Genomes Project Consortium (2015). "A global reference for human genetic variation". In: *Nature* 526.7571, pp. 68–74. DOI: 10.1038/nature15393.

Bercovich, U, MS Rasmussen, Z Li, C Wiuf, and A Albrechtsen (2025). "Measuring linkage disequilibrium and improvement of pruning and clumping in structured populations". In: *Genetics* 229.3, iyaf009. DOI: 10.1093/genetics/iyaf009.

Bulik-Sullivan, BK, PR Loh, HK Finucane, S Ripke, J Yang, et al. (2015). "LD Score regression distinguishes confounding from polygenicity in genome-wide association studies". In: *Nature Genetics* 47.3, pp. 291–295. DOI: 10.1038/ng.3211.

Meurer, A, CP Smith, M Paprocki, O Čertík, SB Kirpichev, et al. (Jan. 2017). "SymPy: symbolic computing in Python". In: *PeerJ Computer Science* 3, e103. DOI: 10.7717/peerj-cs.103.

Ragsdale, AP and S Gravel (2019). "Unbiased Estimation of Linkage Disequilibrium from Unphased Data". In: *Molecular Biology and Evolution* 37.3, pp. 923–932. DOI: 10.1093/molbev/msz265.
